# Neural Representation of Auditory Size in the Human Voice and in Sounds from Other Resonant Sources

**DOI:** 10.1016/j.cub.2007.05.061

**Published:** 2007-07-03

**Authors:** Katharina von Kriegstein, David R.R. Smith, Roy D. Patterson, D. Timothy Ives, Timothy D. Griffiths

**Affiliations:** 1 Wellcome Department of Imaging Neuroscience, Institute of Neurology, University College London, London, WC1N 3BG, United Kingdom; 2 Auditory Group, University of Newcastle upon Tyne Medical School, Newcastle upon Tyne, NE2 4HH, United Kingdom; 3 Department of Psychology, University of Hull, Hull, HU6 7RX, United Kingdom; 4 Centre for the Neural Basis of Hearing, Department of Physiology, Development and Neuroscience, University of Cambridge, Cambridge, CB2 3EG, United Kingdom

**Keywords:** SYSNEURO

## Abstract

The size of a resonant source can be estimated by the acoustic-scale information in the sound [1–3]. Previous studies revealed that posterior superior temporal gyrus (STG) responds to acoustic scale in human speech when it is controlled for spectral-envelope change (unpublished data). Here we investigate whether the STG activity is specific to the processing of acoustic scale in human voice or whether it reflects a generic mechanism for the analysis of acoustic scale in resonant sources. In two functional magnetic resonance imaging (fMRI) experiments, we measured brain activity in response to changes in acoustic scale in different categories of resonant sound (human voice, animal call, and musical instrument). We show that STG is activated bilaterally for spectral-envelope changes in general; it responds to changes in category as well as acoustic scale. Activity in left posterior STG is specific to acoustic scale in human voices and not responsive to acoustic scale in other resonant sources. In contrast, the anterior temporal lobe and intraparietal sulcus are activated by changes in acoustic scale across categories. The results imply that the human voice requires special processing of acoustic scale, whereas the anterior temporal lobe and intraparietal sulcus process auditory size information independent of source category.

## Results and Discussion

In the animal world, the analysis of size from the calls of conspecifics is crucial in social communication (deer [2], frogs [4]). Monkeys can link the size information in conspecific calls to the actual physical appearance of the caller [1]. Humans are also very good at judging size information from auditory cues [3, 5, 6]. This is not only the case for speech stimuli, but also for musical instruments [6]. Furthermore, the perception of acoustic scale does not appear to depend on learned associations based on audio-visual co-occurrence; listeners can extract size information from speech that has been resynthesized to simulate unnatural speaker sizes [3]. This suggests that there is a generic mechanism for the analysis of acoustic scale—a mechanism that supports size perception across a range of natural categories and possibly even in novel categories.

**Figure 1:**
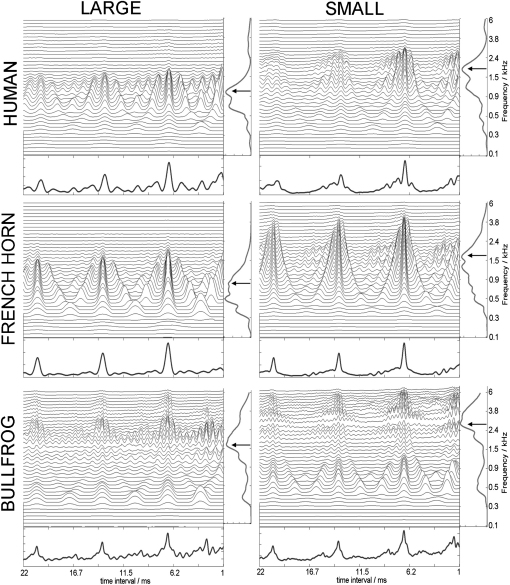
Examples of Auditory Stimuli Used in the Experiment Sounds emitted by a human (the vowel /a/), a French horn (a sustained note), and a bullfrog (a natural croak) are displayed as auditory images [6, 10] with a spectral profile to the right and a temporal profile at the bottom of each panel. For each category, the left-hand column shows a large source and the right-hand column shows a small source. For each category, the reduction in resonator size shifts the distribution of energy up to higher frequencies in the auditory image, and it decreases the duration of the resonances. The auditory image separates the formant and pitch information graphically. The main formant of each sound is marked by a black arrow on the spectral profile to the right of each auditory image. The first peak in the temporal profile (at the bottom of each auditory image) summarizes the pitch information shown by the vertical ridge in the auditory image above the peak in the profile. The sounds in the figure all have the same pitch (147 Hz). When the pitch increases, the peaks move to the right without altering the position of the activity in the frequency dimension.

The size of a resonant source affects the sounds it produces; other things being equal, larger resonant sources vibrate more slowly [7]. In the case of mammals, birds, and frogs, the vocal folds produce a stream of pulses that are filtered by resonances in the vocal tract. The vocal tract grows with body size, and, as a result, the acoustic scale of the sound increases with the length of the vocal tract [8, 9]. In the case of brass instruments, the lips and mouthpiece produce a stream of pulses that are then filtered by resonances in the tube and bell [6]. The effects of the size of the body resonators on the sound are effectively the same for these different sources; the resonant frequencies *increase* in proportion to the *decrease* in the size of the source. The effects of a change in size on a human vowel, a French horn note, and a bullfrog call are illustrated in Figure 1, which presents auditory images of the sounds [6, 10], and in Figure S1 (in the Supplemental Data available online), which presents spectrograms. In the auditory images, the shape of the distribution of activity represents the timbre of the source; the distribution moves upward as a unit (on a log-frequency axis) as the size of the source decreases. The spectral profile to the right of each auditory image emphasizes the timbre of the source in the shape of the profile. The acoustic scale of the source is determined by the position of the profile in frequency. The temporal profile at the bottom of each auditory image emphasizes the pitch of the source [10].

Previous studies revealed that the neural activity associated with the acoustic-scale information in speech sounds is located in posterior superior temporal gyrus (STG) (unpublished data, [11]). The activity was independent of the task the subjects were performing, indicating an obligatory processing step for acoustic-scale information (unpublished data). Here we investigated whether this region in posterior STG is also specialized for acoustic-scale information in other communication sounds. Whereas we previously controlled for spectral-envelope change by varying the speech sounds in all conditions, here we contrast two different types of spectral-envelope change: One of these spectral changes (change of category) changes the shape of the spectral distribution; the other (change of acoustic scale) preserves the shape of the spectral distribution and just changes its position on the frequency axis.

The stimuli for the study were recorded from one speaker (saying /a/), one bullfrog (*Lithobates Catesbeiana*, formerly *Rana catesbeiana*), and one musical instrument (French horn). We analyzed and resynthesized these sounds with a technique that synchronizes the analysis window with the pulse rate of the sound [12, 13]. This allows segregation of the pitch information from the spectral-envelope information that represents the timbre of the source and its size. Once separated, the two forms of information can be manipulated independently and then recombined to simulate variously sized resonant sources with the same pitch (see Supplemental Data for details). Detailed behavioral experiments had been performed with the horn and speech stimuli previously [3, 6, 14]. We performed a perceptual study with the frog sounds to enable us to match the just-noticeable difference (JND) in size across categories (see Supplemental Data for details). Thus, the three categories we used in the two experiments reported here were human speech, animal call, and musical instrument.

Neural activity was assessed with functional magnetic resonance imaging (fMRI). The stimuli were concatenated to form blocks of several sounds. The first experiment was performed as a 2 × 2 factorial design with the factors acoustic scale (fixed, varies) and category (fixed, varies). This resulted in four conditions: (1) acoustic scale varies, category varies; (2) acoustic scale varies, category is fixed; (3) acoustic scale is fixed, category varies; and (4) acoustic scale is fixed, category is fixed (see Experimental Procedures for more details). The first hypothesis was that contrasting conditions in which both types (acoustic scale and category) of spectral envelope vary with those in which both were fixed would reveal areas that are responsive to spectral-envelope change in general. Furthermore, we contrasted the condition in which acoustic scale alone varies with the condition in which category alone varies in order to test the second hypothesis, that there are specific mechanisms—independent of category in posterior STG—for acoustic-scale processing.

**Figure 2:**
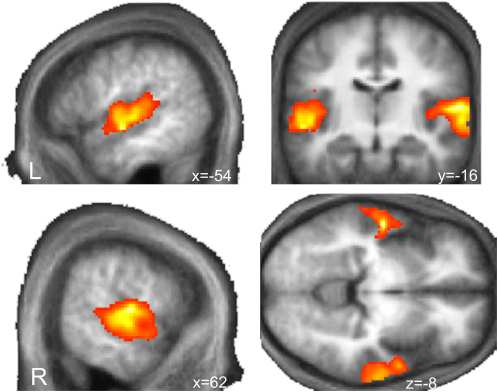
Effect of Changes in Spectral Envelope in Experiment 1 The group (n = 15) statistical parametric map for the contrast between the condition where acoustic scale and category changes and the condition where both are fixed has been rendered on sections of the group mean, normalized structural-MRI volume (p < 0.001, uncorrected).

**Figure 3:**
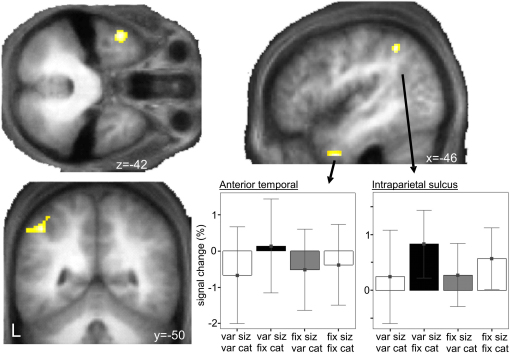
Effect of Acoustic Scale Independent of Category in Experiment 1 The group (n = 15) statistical parametric map for the contrast between the condition where acoustic scale changes (category fixed) and the condition where category is changing (acoustic scale fixed) has been rendered on sections of the group mean, normalized structural-MRI volume (effects are significant at p < 0.001, uncorrected; visualization threshold is p < 0.003). The plots show the parameter estimates at the maximum of activity in the anterior temporal lobe (MNI coordinate: −46, 0, −42) and the intraparietal sulcus (MNI coordinate: −46, −50, 46) for each condition separately. Note that in the changing-size condition, the main spectral peak ranges over 1.5 kHz, whereas the major peak changes by no more than 1.0 kHz in the changing-category condition. The main peak shifts most in the condition where both size and category are changing; this condition includes a change from a large French horn (main peak around 0.5 kHz) to a small frog (main peak at 3 kHz). The plots show that there is more activation in the condition size changing/category fixed than in the condition where both category and size are changing. Therefore, the effects in anterior temporal lobe and intraparietal sulcus cannot be accounted for by the change of the main peak in the spectrum. Black and gray bars represent the conditions that were contrasted against each other (black = +1, gray = −1). The following abbreviations are used: Var, variable; Fix, fixed; Siz, size (acoustic scale); and Cat, Category. Error bars represent 95% confidence interval of the mean.

We localized the auditory system functionally by contrasting all experimental conditions against silence (Figure S2). Areas that are responsive to spectral-envelope change in general were sought by contrasting the condition with acoustic-scale and category changes with the condition where both were fixed. This revealed activity that extended from bilateral STG into planum temporale (PT) and down into the superior temporal sulcus (STS) (Figure 2). To investigate whether acoustic-scale changes in general play a role in the activation of posterior STG, we contrasted the condition where only acoustic scale varies with the condition where only category varies. We did not use the main effect for acoustic scale (Figure S3) to investigate this effect because the perception of size change is relative; if there is a category change within the block, this interferes with the perception of size change. There was no activity in posterior STG (p > 0.05, uncorrected). The contrast revealed activity in left anterior temporal lobe and left intraparietal sulcus (Figure 3).

Anterior-temporal-lobe activity is expected during the higher-level processing of size information because it lies at a site of convergence between processing mechanisms for human visual and auditory communication signals [15]. This does not imply that the anterior temporal area is the only possible site of auditory-visual convergence in size processing. Such convergence (especially during or after bimodal stimulation) probably involves a whole network of interactions from primary to more specialized sensory areas in auditory, as well as visual, cortices [15–17].

Several single-cell studies in animals have shown “numerons” (number-selective neurons) responding to a specific quantity or number independent of its appearance. These are therefore thought to contain an abstract representation of magnitude (for a review, see [18]). Size and magnitude are both continuous dimensions, and comparative judgments are possible in both. Human functional-imaging studies have revealed that both dimensions activate the human intraparietal sulcus [19, 20]. These studies investigated abstract representation of size with visual displays including differently sized letters or numbers. We assume that the activity in intraparietal sulcus in response to auditory size in our study is elicited because the abstract representation of size is accessed not only by the visual but also by the auditory modality.

The fact that size changes in resonant sources do not, in general, activate posterior STG was surprising. In the previous experiments (unpublished data), we did not have conditions where the speech sound was fixed and acoustic scale changed on its own, and vice versa. This might explain the difference because the spectral-envelope changes that code vowel changes were present in all conditions. It could therefore be that the changes in acoustic scale in our previous studies (unpublished data) activate regions implicated in general processing of the spectral envelope. Another possibility is that there is an acoustic-scale-processing region that is specific to the human voice.

The second experiment was designed to test the hypothesis that there is a specific region specialized for processing acoustic-scale information in the human voice. We used the same stimuli as in the first experiment. The difference was in the design, which was a 2 × 3 factorial, with the factor acoustic scale (varies or fixed) and the factor category (animal, musical instrument, human). The three categories were presented in separate blocks, in which the size of the resonant source either varied or was fixed.

**Figure 4:**
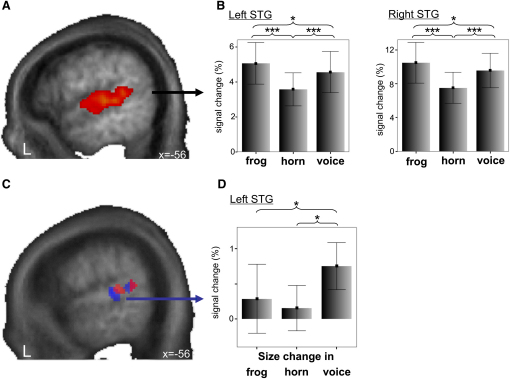
Effects of Category and Acoustic Scale in Experiment 2 The group (n = 14) statistical parametric maps have been rendered on sections of the group mean, normalized structural-MRI volume. (A) Main effect of category. (B) Plot of parameter estimates for each category separately, independent of whether acoustic scale changes or not. (C) Blue shows interaction of acoustic scale and category; red shows conjunction of the main effects of size change in the two previous experiments (unpublished data). (D) Parameter estimates for acoustic scale changing greater than acoustic scale fixed for all source categories separately. All contrasts are significant at p < 0.001, uncorrected; the visualization threshold is p < 0.003. ^∗^ indicates p < 0.05. ^∗∗∗^ indicates p < 0.001. Error bars represent 95% confidence interval of the mean.

The main effect of category revealed activity in STG and STS (Figure 4A). This activity was mostly accounted for by differential activation to frog and human sounds when contrasted with horn sounds (Figure 4B). The interaction of acoustic scale and category (Figure 4C, blue) is significant in the left STG. This activity overlaps with activity found in previous experiments (unpublished data) for size changes in speech (Figure 4C, red). The parameter estimates show that the interaction of acoustic scale and category in the present study is due to the strength of the signal change associated with the size information in the human voice (Figure 4D). This contrast is controlled for activity in response to spectral-envelope change in general because it contrasts the difference between varying and fixed acoustic scale in the human voice against the difference between varying and fixed acoustic scale in the other categories. Furthermore, the differential activity for size change in vowel sounds cannot be attributed to greater activity for voices in general: The category effect shows that frog sounds activate the STG to a higher extent than vowel sounds (Figure 4B).

These results are consistent with the hypothesis that there is a computational mechanism within posterior STG for the analysis of acoustic scale in the human voice. This is not, however, a generic mechanism for the analysis of size in resonant sources. The posterior superior temporal lobe has been implicated as a primary substrate for constructing sound-based representations of speech [21]. Differential activation to a vowel sound would be reasonable inasmuch as formant shifts associated with size differences need to be separated from formant shifts associated with linguistic content only in human communication. Normalization for acoustic scale is not required to the same degree for the communication calls of other species. Another possible explanation would be that activity in posterior STG is related to the number of formants that change size, a number that is larger for human speech.

In summary, our results show that the activity in posterior STG found for acoustic scale in previous studies (unpublished data) reflects processing of acoustic scale specifically for human speech. In contrast, activity in the anterior temporal lobe appears to reflect general processing of acoustic scale. This region constitutes a candidate area for higher-level processing of size information, such as that involved in audio-visual matching of the sound of the source with its physical appearance [15]. Furthermore, category-independent activity to acoustic scale in intraparietal sulcus supports the view that intraparietal sulcus is not only accessed by size judgements on visual stimuli [19, 20], but also entails a general supramodal representation of size.

## Experimental Procedures

### Subjects

Fifteen subjects participated in experiment 1 and 14 in experiment 2. For further details, see the Supplemental Data.

### Stimuli

There were three categories of tonal sounds in these experiments: the vowel /a/, a bullfrog croak, and a French-horn note. Each category was constructed from a single prototype sound by manipulation of the fundamental frequency (f0) of the prototype sound and the acoustic scale of the envelope of the sound with the vocoder, STRAIGHT [12, 13]. For further details see Supplemental Data.

### Experimental Design

Sets of stimuli were concatenated to form the stimulus block for a trial. For monitoring of alertness, subjects pressed a button at the end of each block.

#### Experiment 1

The design included the four experimental conditions described above and one silent condition. There were three categories (frogs, horns, humans) and three acoustic-scale values (ac2, ac5, ac10). Twelve sounds were concatenated to form a block for one trial. In the conditions where acoustic scale or category was fixed, the scale or category was chosen randomly from the three possible values, and it was fixed during the 12 sounds of the trial. In the conditions where acoustic scale or category (or both) varied, the block still consisted of 12 sounds; however, the scale or category varied (or both varied).

#### Experiment 2

The design included the six experimental conditions and one silent condition. Each of the three stimulus categories (frogs, horns, humans) was represented by a condition with fixed acoustic scale and a condition with varying acoustic scale. There were eleven possible acoustic-scale values in the experiment. The block for a trial consisted of 11 concatenated sounds. In the fixed-acoustic-scale conditions, a scan consisted of one sound repeated 11 times. The acoustic-scale value for a fixed condition was chosen randomly for each scan from the 11 possible values. In the varied-scale conditions, the category was fixed throughout the block of 11 sounds, while the acoustic scale varied randomly from sound to sound, with the restriction that the value must be two or more steps away from the preceding value. This randomization ensured that each stimulus was at least 4 JNDs from its temporal neighbors, and normally more than 4 JNDs.

The pitch value in both experiments was kept fixed within a trial, and the value was chosen randomly from the 30 possible values.

### Scanning Procedure

The stimuli were delivered via a custom electrostatic system at 70 dB sound-pressure level (SPL). Each sound sequence was presented for a period of 9.2 s (experiment 1) or 8.4 s (experiment 2), after which brain activity was estimated by the fMRI blood-oxygen-level-dependent (BOLD) response at 3 T (Siemens Allegra, Erlangen, Germany) by gradient-echo-planar imaging in a sparse acquisition protocol [22, 23] with cardiac gating (time to repeat/time to echo [TR/TE]: 2.73 s + length of stimulus presentation/65 ms; 42 transverse slices; 3 × 3 mm in-plane resolution, ascending axial sequence). Two hundred and fifty-two brain volumes/subject (48 volumes/condition) were acquired for experiment 1 and 240 brain volumes/subject (30 volumes/experimental condition, 60 volumes/silence condition) for experiment 2.

### Image Analysis

Imaging data were analyzed with statistical parametric mapping implemented in SPM2/5 software (http://www.fil.ion.ucl.ac.uk/spm/). Scans were realigned, unwarped, spatially normalized [24] to Montreal Neurological Institute (MNI) standard stereotactic space [25], and spatially smoothed (8 mm full-width-at-half-maximum).

Statistical parametric maps were generated by modeling the evoked hemodynamic response as boxcars convolved with a synthetic hemodynamic-response function in the context of the general linear model [26]. Population-level inferences concerning BOLD signal changes between conditions of interest were based on a random-effects model. For each contrast, responses were considered significant at p < 0.001, uncorrected, if the localization of activity was in accordance with prior hypothesis.

## Supplemental Data

Document S1. Experimental Procedures and Three Figures
