# Supplementary material for: Neural Representation of Auditory Size in the Human Voice and in Sounds from Other Resonant Sources
Source: Curr Biol. 2007 Jul 3;17(13-2):1123–8. doi: 10.1016/j.cub.2007.05.061 (PMC2335591; doi:10.1016/j.cub.2007.05.061)
Supplement: Document S1. Experimental Procedures and Three Figures [file mmc1.pdf]

Supplemental Data

Neural Representation of Auditory Size in the Human Voice and in Sounds from Other Resonant Sources

Katharina von Kriegstein, David R.R. Smith, Roy D. Patterson, D. Timothy Ives, and Timothy D. Griffiths

Supplemental Experimental Procedures

Subjects

All subjects in both experiments were right handed (experiment 1: 8 female, 7 male, aged 22–32 yr, mean 27; experiment 2: 8 female, 6 male, aged 19–40 yr, mean 26). All subjects gave informed consent, and the experiment was carried out with the approval of the Institute of Neurology Ethics Committee, London. No subject had any history of audiological or neurological disorder, and all had normal structural-MRI brain scans.

Stimuli

STRAIGHT is a sophisticated software package that performs a pitch-synchronous spectral analysis of each sound and provides an f0 contour and a pitch-independent spectrogram that describes the filtering action of the source. Once segregated, the two forms of information can be manipulated independently, and sounds from one source (human, bullfrog, or horn) can be synthesized with varying f0 and acoustic scale. Example sounds are provided at http://www.pdn.cam.ac.uk/groups/cnbh/index.html. All of the stimuli in all of the categories were resynthesized with 16 bit resolution, a 48 kHz sampling rate, a 683 ms duration, and a fixed root mean square (RMS) level.

The positions of the sounds within their files were adjusted such that their perceptual centers (P centers) all occurred at the same time relative to the file onset. This P center correction [S1] ensured that when any combination of the sounds was played in a sequence, they would be perceived to proceed at a regular pace; an irregular sequence of sounds causes an unwanted distraction. The average P centers, before correction, were 169 ms, 326 ms, and 189 ms for the speech, bullfrog, and horn stimuli, respectively.

Speech sounds

The prototype vowel was an /a/ drawn from a custom speech database at the Centre for Neural Basis of Hearing [S1, S2]. The speech category was restricted to a single canonical token of /a/ to ensure that the category did not provide more stimulus variability than the animal and musical categories, which were restricted by a lack of natural variation. The /a/ was resynthesized with one of thirty f0s, ranging from 95 Hz to 220 Hz in logarithmic (quartertone) steps. The acoustic scale of the STRAIGHT spectrogram was varied over a similar range, from 75% to 188% of the scale of the prototype sound, in ten equal, logarithmic steps (ac1–ac10). The ends of this range correspond to the sounds produced by very large men and small children, respectively [S3]. The just-noticeable difference (JND) for discriminating a change in the acoustic scale of speech sounds is approximately 6% [S1, S4], so each logarithmic step represents approximately 2 JNDs.

Bullfrog Calls

The prototype bullfrog call (*Lithobates catesbeiana*) was drawn from a database of male territorial calls described in [S5]. The fundamental of the call is about 110 Hz, so it was relatively easy to resynthesize the sounds with the same f0 values as for the speech stimuli. The acoustic scale of the STRAIGHT spectrogram was varied over the same range as for the speech stimuli, in this case from 55% to 138% of the scale of the prototype bullfrog call, in ten equal, logarithmic steps (ac1–ac10). Pilot work indicated that for human listeners, the JND for envelope scale was about the same for bullfrog calls as for speech stimuli, so each step represents approximately 2 JNDs.

French-Horn Notes

The prototype French-horn note was drawn from a database of musical sounds [S6, S7]. The sounds were resynthesized with the same f0 values as for the vowels and bullfrog calls. The acoustic scale of the STRAIGHT spectrogram was varied over the same range as for the speech stimuli, in this case from 40% to 159% of the prototype horn note, in ten equal, logarithmic steps (ac1–ac10). The JND for acoustic scale in horn sounds is approximately 7% [S6], so once again, each step represents approximately 2 JNDs.

Supplemental References

S1. Ives, D.T., Smith, D.R.R., and Patterson, R.D. (2005). Discrimination of speaker sizes from syllable phrases. J. Acoust. Soc. Am. *118*, 3816–3822.

S2. von Kriegstein, K., Warren, J.D., Ives, D.T., Patterson, R.D., and Griffiths, T.D. (2006). Processing the acoustic effect of size in speech sounds. Neuroimage *32*, 368–375.

S3. Fant, G. (1970). Acoustic Theory of Speech Production, Second Edition (Paris: Mouton).

S4. Smith, D.R.R., Patterson, R.D., Turner, R., Kawahara, H., and Irino, T. (2005). The processing and perception of size information in speech sounds. J. Acoust. Soc. Am. *117*, 305–318.

S5. Bee, M.A. (2004). Within-individual variation in bullfrog vocalizations: implications for a vocally mediated social recognition system. J. Acoust. Soc. Am. *116*, 3770–3781.

S6. van Dinther, R., and Patterson, R.D. (2006). The perception of size in musical instruments. J. Acoust. Soc. Am. *120*, 2158–2176.

S7. Goto, M., Hashiguchi, H., Ishimura, T., and Ka, R. (2003). RWC music database: music genre database and musical instrument databse. Proceedings of the Fourth International Conference on Music Information Retrieval (ISMIR), 229–230.

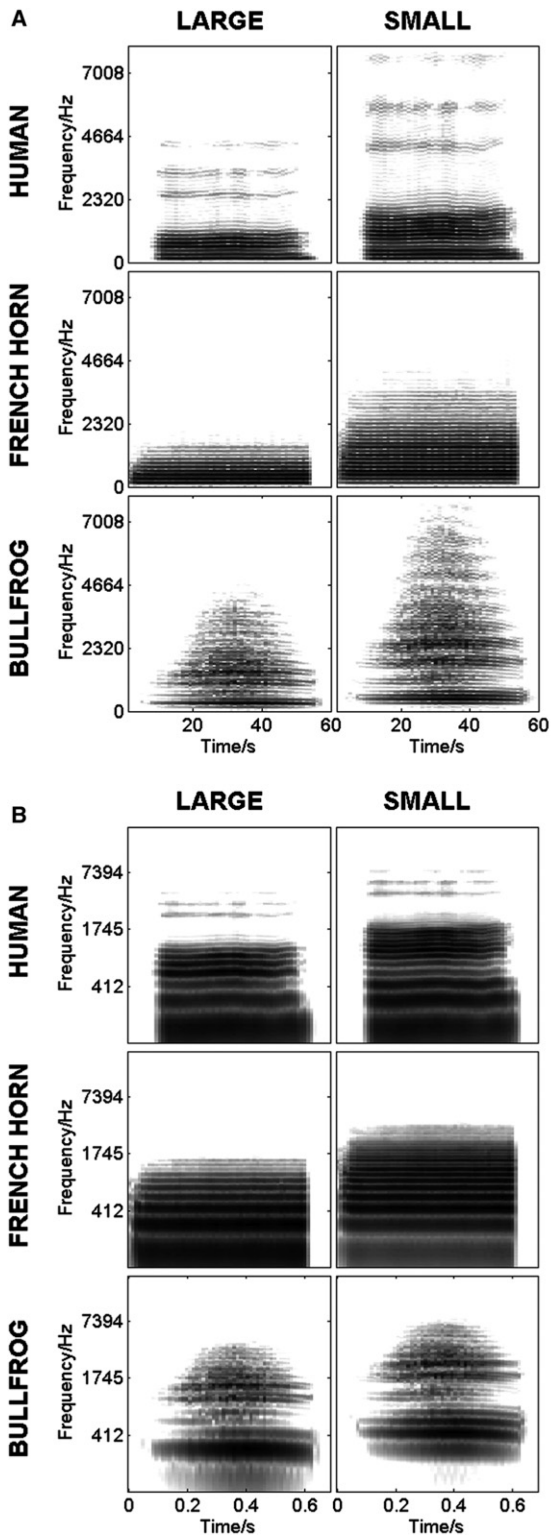

Figure S1. Spectrograms for the Stimuli Displayed in Figure 1

The vowel /a/, the French-horn note, and the bullfrog croak are displayed with the traditional linear-frequency axis (A) and an alternative logarithmic-frequency axis (B). For each stimulus category in both (A) and (B), the left-hand column shows a large source and the right-hand column shows a small source. For each category, the reduction in resonator size causes an expansion of the distribution of energy to higher frequencies: The expansion is emphasized in the traditional narrow-band spectrogram (A) with its linear-frequency axis. The distribution shifts as a unit without dilation in the log-frequency spectrogram (B); in this case, the position of the distribution represents resonator size separate from the shape of the distribution. The glottal pulses that define the pitch are removed by the temporal integration used to produce the spectrograms.

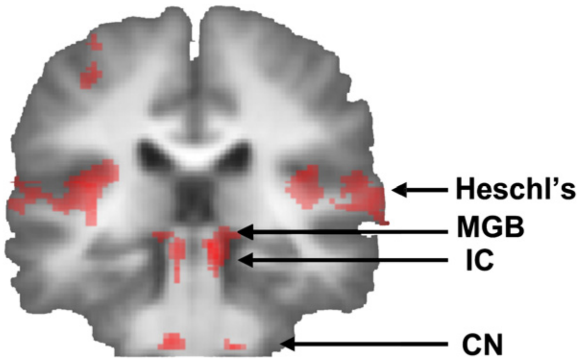

Figure S2. Mapping the Auditory System with the Contrast between All Experimental Conditions and Silence

The sounds activate all of the subcortical auditory nuclei along with the main auditory regions in cortex. The group statistical parametric map for the contrast has been rendered on a coronal section of the group mean, normalized structural MRI volume. The contrast was performed in both experiments. This figure shows the contrast for experiment 2. CN denotes cochlear nucleus, IC denotes inferior colliculus; and MGB denotes medial geniculate body.

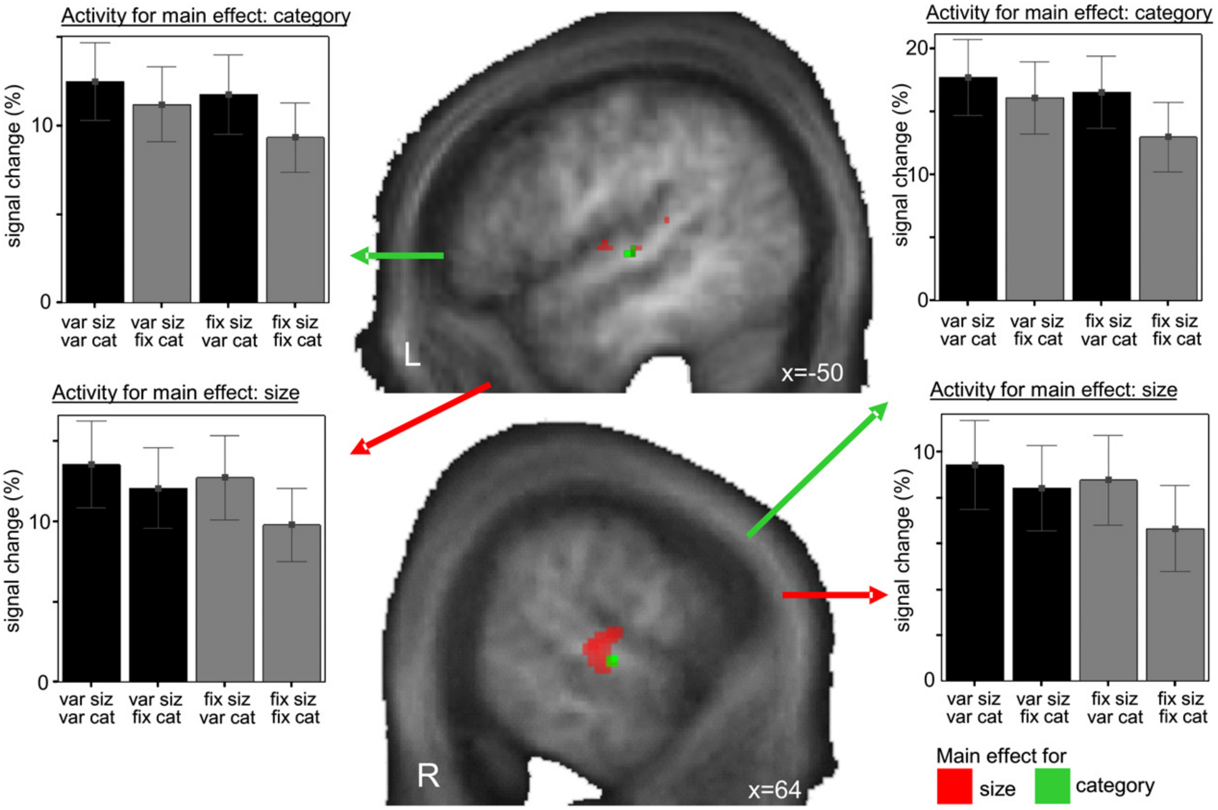

Figure S3. Main Effects in Experiment 1

Main effect of size (red) and category (green). The main effect of size, as well as the main effect of category, revealed activity in bilateral superior temporal gyrus (visualization here at $p < 0.05$ family-wise error [FWE] corrected). Plotting the parameter estimates for each condition separately revealed that the activity is not specific to the spectral-envelope changes in size: There was no difference between activity for the condition with varying acoustic scale (category fixed) in contrast to the condition with varying category (acoustic scale fixed). Also, the area previously responsive to size in speech (controlled for spectral-envelope change) did not show any differential activation. Black and gray bars represent the conditions that were contrasted against each other (black = +1, gray = −1). Error bars represent 95% confidence interval of the mean.
